# Bone marrow stromal cells show distinct gene expression patterns depending on symptomatically involved organs in multiple myeloma

**DOI:** 10.1038/bcj.2016.86

**Published:** 2016-09-23

**Authors:** S Y Kim, K Im, S N Park, B Oh, J-A Kim, S M Hwang, M Kim, S-S Yoon, D S Lee

**Affiliations:** 1 Department of Laboratory Medicine, Seoul National University College of Medicine, Seoul, Republic of Korea; 2 Department of Laboratory Medicine, Chungnam National University School of Medicine, Daejeon, Republic of Korea; 3 Cancer Research Institute, Seoul National University College of Medicine, Seoul, Republic of Korea; 4 Department of Oncology, Asan Medical Center, Seoul, Republic of Korea; 5 Department of Laboratory Medicine, Seoul National University Bundang Hospital, Seongnam, Republic of Korea; 6 Department of Laboratory Medicine, Hallym University Sacred Heart Hospital, Anyang, Republic of Korea; 7 Department of Internal Medicine, Seoul National University College of Medicine, Seoul, Republic of Korea

Diagnosis of symptomatic multiple myeloma (MM) is made only when myeloma-related symptoms or end-organ damage occur, including hypercalcemia, renal insufficiency, anemia and bone lesions (CRAB).^1^ Little is known at present as to why the clonal proliferation of plasma cells exhibits different organ involvement among myeloma patients, for example, bone lesion vs. renal failure. For the most part, bone disease in myeloma is thought to result from the overexpression of the receptor activator of NF-κB ligand by bone marrow (BM) stroma, which in turn activates osteoclasts. On the other hand, the pathology of renal disease in myeloma is heterogeneous and may involve a variety of mechanisms.^2^ It has been suggested that the amount and biochemical characteristics of light chains are important determinants of renal disease presentations in myeloma.^2^ The formation of reactive oxygen species has also been suggested as a potential mechanism of light chain-induced renal injury.

MM is a unique neoplasm, in that clonal plasma cells coexist among normal hematopoietic cells, with gradual predominant growth and ultimate organ damage caused by deposited immunoglobulin. During this process, BM stromal cells presumably contribute to this growth privilege of plasma cells and the determination of which organs will be damaged.^3^ At present, few studies have demonstrated the distinct gene expression profiles of microenvironmental cells in myeloma compared with normal samples.^4, 5, 6^ On the basis of the hypothesis that BM stromal cells play a major role in the involvement of different organs in myeloma, we compared gene expression profiles according to organ involvement after the culture and expansion of BM stromal cells from MM patients with different organ involvement.

We cultured BM stromal cells collected from 11 newly diagnosed MM patients (Supplementary Table S1). One patient presented as asymptomatic MM, and the remaining 10 MM patients were classified according to their myeloma-defining events (MDEs). Bone lesions were mainly detected by complete skeletal survey, and renal function was assessed by serial measurement of creatinine levels (detailed methods are described in Supplementary Information). Six patients presented with multiple lytic bone lesions (bone lesion group): five patients without renal failure and one patient (Patient 3) with transient renal impairment. Three patients presented with renal failure with creatinine clearance <30 ml min^−1^ at diagnosis and <20 ml min^−1^ at the end of follow-up (renal failure group) in the absence of bone disease, and one patient presented with anemia (hemoglobin 8.9 gdl^−1^) without bone lesions and without renal failure (anemia group). For all 5 patients without bone lesions, ⩾2 imaging analyses were performed, either by different methods in addition to complete skeletal survey (Patient 10, computed tomography; and Patient 11, magnetic resonance imaging) or with ⩾2 skeletal surveys at different times (Patients 1, 8 and 9, at diagnosis and at disease progression).

In addition, we cultured BM stromal cells from patients with other plasma cell neoplasms (Supplementary Table S2). One patient (Patient 12) had two bone plasmacytomas in the spine and femur without other symptoms and <10% plasma cells in BM (plasmacytoma group). Two patients were diagnosed with AL amyloidosis (one with cardiac amyloidosis and the other with renal amyloidosis), and one patient was diagnosed with POEMS syndrome. The control groups were composed of nine B-cell lymphoma patients with no evidence of BM involvement and four patients with mild-to-moderate cytopenia without evidence of hematologic malignancies.

Cultured BM stromal cells were analyzed after passage five for all patients. The passages at analysis and growth rates of BM stromal cells varied among patients; however, there were no differences between disease and control groups (Supplementary Table S3). Flow cytometric analysis of BM stromal cells from the MM (*n*=4) and control patients (*n*=5) did not indicate the expression of hematopoietic lineage antigens but did indicate the positive expression of CD90, CD105 and CD44. Cytogenetic studies using G-banding and interphase fluorescence *in situ* hybridization (FISH) to detect common chromosomal abnormalities known to frequently occur in MM was performed on patients' BM aspiration specimens and cultured BM stromal cells (Supplementary Table S4). Eight patients exhibited FISH abnormalities in BM plasma cells, including *IGH* translocations (*n*=6), *RB1* deletions (*n*=5), 1q duplications (*n*=5) and trisomies (*n*=3). *IGH* translocations were detected in 3/6 patients with bone lesions (50%) and in 2/3 patients with renal failure (67%). We tested for BM stromal cells from 10 MM patients using the same FISH probes; however, none of the patients presented the cytogenetic abnormalities observed in the corresponding malignant plasma cells.

When the global gene expression profiles of BM stromal cells from 11 MM patients were compared with those of stromal cells from the 13 control patients, the patient and control groups did not form clearly separated clusters. Otherwise, the patient-derived BM stromal cells exhibited preferential grouping into several main clusters according to clinical manifestations. The gene expression profiles of BM stromal cells from patients with multiple lytic bone lesions and those from patients with renal failure presented as different clusters (Figure 1a). Among the differentially expressed genes in BM stromal cells from MM patients with multiple bone lesions, vascular cell adhesion molecule 1 (*VCAM1*) was overexpressed 3.27-fold. In addition, extracellular matrix genes, such as collagen type IV (*COL4A4*), periostin (*POSTIN*), fibulins (*FBLN2* and *FBLN5*) and secreted frizzled-related protein 4 (*SFRP4*), were overexpressed (Table 1). Differentially expressed genes in BM stromal cells from MM patients with renal failure were associated with cell signaling (*RGS17*, *STEAP1*). The qPCR results presented higher relative expression levels for the *VCAM1* gene in MM with bone lesions (Figure 1b). In the gene set enrichment analysis, the enriched gene sets of BM stromal cells from MM patients with bone lesions were involved in the cellular regions and biological processes of the extracellular matrix (Figure 1c). MM patients with renal failure presented with enhanced expression of gene sets associated with G-protein coupled receptor signaling and the biological process of transmembrane transporter activity (Figure 1d).

In previous studies, distinct gene expression profiles in MM BM mesenchymal stem cells (MSCs) has been reported.^4^ In a previous study, >140 genes were differentially expressed between MM and normal MSCs. Especially, the overexpression of *GDP15* was emphasized as an important genetic alteration in MM BM-MSCs.^4^ In our study, the gene expression profiles of BM stromal cells were not clearly separated between control and myeloma patients, which was contrary to our expectations. We infer that individual variations in stromal cells could have masked pattern detection. Meanwhile, comparing the gene expression profiles of MM patients with bone lesions and patients with renal failure revealed that the profiles were clearly separated. Compared with the extensive information on the gene expression profiles of clonal plasma cells, insufficient MSC data exists to reach informative conclusions. Still, no report have explored the different characteristics of stromal cells based on the involved organs in myeloma. Our results revealed different gene expression patterns between MM patients with bone lesions and MM patients with renal failure, which may suggest a role for stromal cells in determining organ damage in symptomatic MM.

An interesting result of this study is that the highly expressed genes and gene sets present plausible relationships between the MDEs of symptomatic MM and other plasma cell neoplasms. First, MM patients with bone lesions showed elevated expression levels of extracellular matrix-associated genes, such as *COL4A4, POSTN, FBLN2, FBLN5*, *SFRP4* and *VCAM1*. VCAM1 is an adhesion molecule that binds with high affinity to integrin α4β1 and is constitutively expressed in BM stromal cells.^7^ The binding of myeloma cells to BM-MSCs through α4β1-integrin and VCAM1 interaction induces the decreased secretion of osteoprotegerin and the increased expression of receptor activator of NF-κB ligand, which promotes osteolysis.^8, 9^ The profiles of myeloma with bone lesions were rather homogenous, whereas those with renal failure were heterogeneous. This finding is consistent with past observations that myeloma with renal involvement shows a heterogeneous pathology involving a variety of mechanisms.^2, 10^

When the results of other plasma cell neoplasms were analyzed, notch 2 N-terminal-like (*NOTCH2NL*) expression was high in patients with plasmacytoma (Supplementary Figure S1). In myeloma cells, NOTCH signaling is thouhgt to be involved in BM homing, MM cell metastasis, and matrix invasion.^11^ In a patient with POEMS syndrome, stathmin-like 2 (*STMN2*), which encodes the microtubule regulatory protein stathmin, was found to be overexpressed. Interestingly, stathmin is reported to be associated with the expression of VEGF and hypoxia-inducible factor a (HIF-1a).^12^ VEGF is well known as an important cytokine in the pathogenesis of POEMS syndrome.^13^ In AL amyloidosis, the expression of the lambda light chain (*IGLL1*) gene was significantly elevated; however, we could not confirm this result via qPCR (Supplementary Figure S1). Although these results might offer interesting clues, with data from one or two patients, it was not possible to determine whether the genetic variations were disease subtype-specific or patient-specific. More patients with POEMS syndrome or AL amyloidosis should be included.

The correlation between cytogenetic abnormalities in plasma cells and MDEs of MM patients has been reported.^1^ In the present study, we found no specific correlation between chromosomal abnormalities and MDEs. Considering our results of varying gene expression patterns in BM stromal cells among MM patients with different MDEs, we suggest that the microenvironment, in addition to the malignant plasma cells themselves, is also an important factor in determining the clinical symptoms of MM patients.

The small number of patients used is a limitation of this study; therefore, it is difficult to draw definitive conclusions. To confirm the role of stromal cells in determining organ damage, data from a larger study population that encompasses different ethnic groups, as well as *in vitro* and *in vivo* functional studies, will be needed. In addition, the expression profiles were investigated beyond the fifth passage of BM stromal cells; therefore, *in vitro* culture conditions could have affected the gene expression characteristics of the BM stromal cells. However, these results may provide novel information that was absent from previous studies that investigated BM stromal cells at very early passages. For some patients, only complete radiologic skeletal surveys were performed to detect bone lesions. Approximately 10–20% of bone lesions are known to be missed by conventional radiology.^14^ Therefore, with the use of more sensitive techniques, bone lesions may be detected in more patients. Notably, as described above, for the three patients without bone lesions, two complete skeletal surveys were performed at diagnosis and at disease progression. Repeated testing may increase sensitivity compared conventional radiologic bone survey alone, thereby reducing the likelihood that bone lesions might be missed.

In conclusion, the gene expression profiles of BM stromal cells in MM patients differed among patients with different organ involvement, and we hypothesize that differentially expressed genes in stromal cells could play important roles in symptomatic myeloma. Considering that different patients with the same disease could have variable clinical manifestations, organ involvement and disease course, our observations provide an alternative explanation for individual patient variability other than tumor cell heterogeneity.

## Figures and Tables

**Figure 1:**
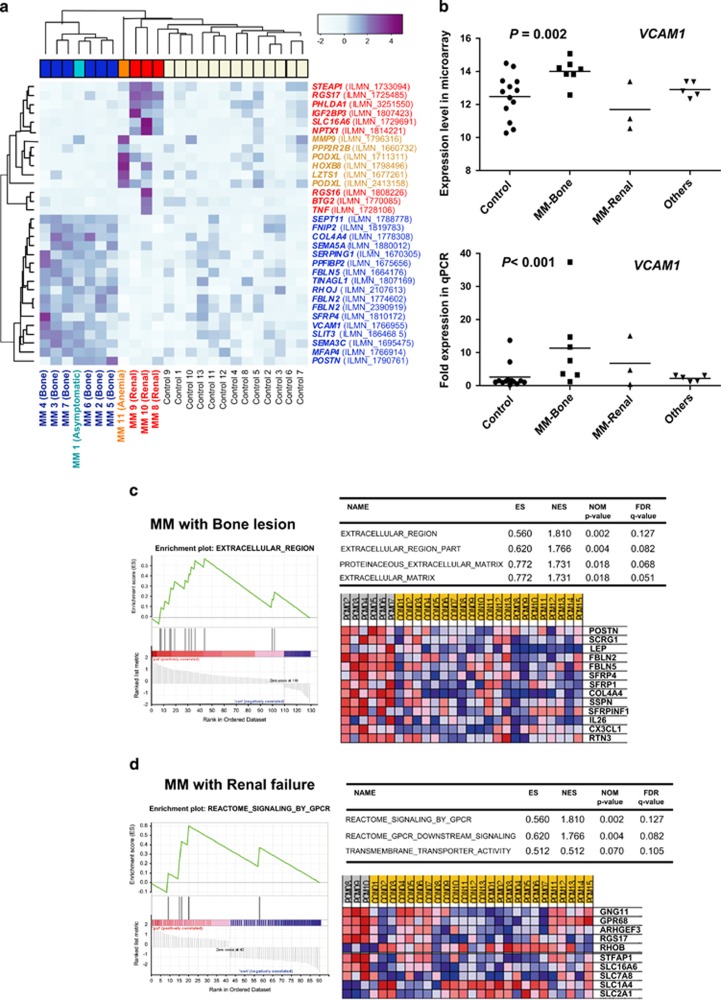
(**a**) Gene expression profiles of bone marrow stromal cells from multiple myeloma (MM) patients according to clinical manifestations. (**b**) Gene expression levels of *VCAM1*, which were estimated by microarray and qPCR assays. Gene set enrichment analysis of bone marrow stromal cells in (**c**) MM patients with bone lesions and in (**d**) MM patients with renal failure.

**Table 1:** Overexpressed genes of BM stromal cells from MM patients with different clinical characteristics

*Gene symbol*	*Entrez ID*	*Score (d)*	*Fold change*	*Chromosome*	*Description*
*MM with bone disease*					
* COL4A4*	1286	4.193	1.77	2q36.3	Collagen, type IV, alpha 4
* SEMA3C*	10512	4.089	2.18	7q21.11	Sema domain, immunoglobulin domain, short basic domain, secreted, (semaphorin) 3C
* VCAM1*	7412	3.961	3.27	1p21.2	Vascular cell adhesion molecule 1
* SEPT11*	55752	3.942	1.94	4q21.1	Septin 11
* PPFIBP2*	8495	3.518	1.94	11p15.4	PTPRF-interacting protein, binding protein 2 (liprin beta 2)
* POSTN*	10631	3.430	2.62	13q13.3	Periostin, osteoblast specific factor
* FBLN2*	2199	3.366	2.72	3p25.1	Fibulin 2
* SERPING1*	710	3.362	2.00	11q12.1	Serpin peptidase inhibitor, clade G (C1 inhibitor), member 1
* FBLN5*	10516	3.324	2.12	14q32.12	Fibulin 5
* FNIP2*	57600	3.287	1.78	4q32.1	Folliculin-interacting protein 2
* ITGBL1*	9358	3.165	1.89	13q33.1	Integrin, beta-like 1 (with EGF-like repeat domains)
* SLIT3*	6586	3.138	2.54	5q35.1	Multiple epidermal growth factor-like domains protein
* MFAP4*	4239	3.061	3.24	17p11.2	Microfibrillar-associated protein 4
* LEPR*	3953	2.993	3.62	1p31.3	Leptin receptor
* SFRP4*	6424	2.365	2.10	7p14.1	Secreted frizzled-related protein 4
					
*MM with renal impairment*					
* NPTX1*	4884	6.665	3.93	17q25.3	Neuronal pentraxin I
* RGS17*	26575	6.168	2.01	6q25.2	Regulator of G-protein signaling 17
* SLC16A6*	9120	4.634	2.49	17q24.2	Solute carrier family 16, member 6 (monocarboxylic acid transporter 7)
* STEAP1*	26872	4.232	3.28	7q21.13	Six transmembrane epithelial antigen of the prostate 1
* UBE2E3*	10477	3.997	1.94	2q31.3	Ubiquitin-conjugating enzyme E2E 3 (UBC4/5 homolog, yeast)

Abbreviations: BM, bone marrow; MM, multiple myeloma.

## References

[bib1] Greenberg AJ, Rajkumar SV, Therneau TM, Singh PP, Dispenzieri A, Kumar SK. Relationship between initial clinical presentation and the molecular cytogenetic classification of myeloma. Leuk: Off J Leuk Soc Am 2014; 28: 398–403. 10.1038/leu.2013.258 PMC3924716 24005246

[bib2] Heher EC, Goes NB, Spitzer TR, Raje NS, Humphreys BD, Anderson KC et al. Kidney disease associated with plasma cell dyscrasias. Blood 2010; 116: 1397–1404. 20462963 10.1182/blood-2010-03-258608 PMC3324369

[bib3] Raab MS, Podar K, Breitkreutz I, Richardson PG, Anderson KC. Multiple myeloma. Lancet 2009; 374: 324–339. 19541364 10.1016/S0140-6736(09)60221-X

[bib4] Corre J, Mahtouk K, Attal M, Gadelorge M, Huynh A, Fleury-Cappellesso S et al. Bone marrow mesenchymal stem cells are abnormal in multiple myeloma. Leuk: Off J Leuk Soc Am 2007; 21: 1079–1088. 10.1038/sj.leu.2404621 PMC2346535 17344918

[bib5] Todoerti K, Lisignoli G, Storti P, Agnelli L, Novara F, Manferdini C et al. Distinct transcriptional profiles characterize bone microenvironment mesenchymal cells rather than osteoblasts in relationship with multiple myeloma bone disease. Exp Hematol 2010; 38: 141–153. 19963035 10.1016/j.exphem.2009.11.009

[bib6] Ria R, Todoerti K, Berardi S, Coluccia AM, De Luisi A, Mattioli M et al. Gene expression profiling of bone marrow endothelial cells in patients with multiple myeloma. Clin Cancer Res: Off J Am Assoc Cancer Res 2009; 15: 5369–5378. 10.1158/1078-0432.CCR-09-0040 19690192

[bib7] Chen Q, Massague J. Molecular pathways: VCAM-1 as a potential therapeutic target in metastasis. Clin Cancer Res: Off J Am Assoc for Cancer Res 2012; 18: 5520–5525. 10.1158/1078-0432.CCR-11-2904 PMC3473104 22879387

[bib8] Michigami T, Shimizu N, Williams PJ, Niewolna M, Dallas SL, Mundy GR et al. Cell-cell contact between marrow stromal cells and myeloma cells via VCAM-1 and alpha(4)beta(1)-integrin enhances production of osteoclast-stimulating activity. Blood 2000; 96: 1953–1960. 10961900

[bib9] Hideshima T, Mitsiades C, Tonon G, Richardson PG, Anderson KC. Understanding multiple myeloma pathogenesis in the bone marrow to identify new therapeutic targets. Nat Rev Cancer 2007; 7: 585–598. 17646864 10.1038/nrc2189

[bib10] Merlini G, Pozzi C. Mechanisms of renal damage in plasma cell dyscrasias: an overview. Contrib Nephrol 2007; 153: 66–86. 17075224 10.1159/000096761

[bib11] Colombo M, Mirandola L, Platonova N, Apicella L, Basile A, Figueroa AJ et al. Notch-directed microenvironment reprogramming in myeloma: a single path to multiple outcomes. Leuk: Off J Leuk Soc Am 2013; 27: 1009–1018. 10.1038/leu.2013.6 23307030

[bib12] Yoshie M, Miyajima E, Kyo S, Tamura K. Stathmin, a microtubule regulatory protein, is associated with hypoxia-inducible factor-1alpha levels in human endometrial and endothelial cells. Endocrinology 2009; 150: 2413–2418. 19179443 10.1210/en.2008-1333

[bib13] Dispenzieri A. POEMS syndrome: 2014 update on diagnosis, risk-stratification, and management. Am J Hematol 2014; 89: 214–223. 24532337 10.1002/ajh.23644

[bib14] Dimopoulos M, Terpos E, Comenzo RL, Tosi P, Beksac M, Sezer O et al. International myeloma working group consensus statement and guidelines regarding the current role of imaging techniques in the diagnosis and monitoring of multiple Myeloma. Leuk: Off J Leuk Soc Am 2009; 23: 1545–1556. 10.1038/leu.2009.89 19421229

