# Supplementary material for: Bone marrow stromal cells show distinct gene expression patterns depending on symptomatically involved organs in multiple myeloma
Source: Blood Cancer J. 2016 Sep 23;6(9):e476–. doi: 10.1038/bcj.2016.86 (PMC5056976; doi:10.1038/bcj.2016.86)
Supplement: Supplementary Information [file bcj201686x1.doc]

**SUPPLEMENTARY INFORMATION**

**Bone marrow stromal cells show distinct gene expression patterns depending on symptomatically involved organs in multiple myeloma**

**Supplemental Methods**

**Patients**

Study data were collected from 11 newly diagnosed multiple myeloma (MM) patients treated at Seoul National University Hospital. Patient characteristics are presented in supplemental Table S1. Eleven patients were diagnosed as MM with increased monoclonal proteins and ≥ 10% plasma cell content in the bone marrow (BM). One patient presented as asymptomatic MM, and the remaining 10 MM patients were classified according to their clinical presentation, as defined by myeloma-defining events (MDEs). To detect bone lesions, complete radiologic skeletal survey was performed in each patient. For some patients, additional computed tomography (CT) or magnetic resonance imaging (MRI) was performed. To assess renal function, creatinine clearance was calculated each patient at diagnosis. Chronic renal failure and transient renal impairment were distinguished using serial measurement of creatinine levels. Six patients presented with multiple lytic bone lesions (bone lesion group): five patients without renal failure and one patient (Patient 3) with transient renal impairment. Three patients presented with renal failure with creatinine clearance < 30 mL/min at diagnosis and < 20 mL/min at the end of follow-up (renal failure group) in the absence of bone disease, and one patient presented with anemia (hemoglobin 8.9 g/dL) without bone lesions and without renal failure (anemia group). For all 5 patients without bone lesions, more than 2 imaging analyses were performed either by different methods or with single method at multiple times. For 3 patients (Patients 1, 8, and 9), two complete skeletal surveys were performed: once at diagnosis and again at disease progression at approximately 2 years after diagnosis. For Patient 10, radiologic skeletal survey and CT were performed at diagnosis. For Patient 11, skeletal survey and MRI were performed at diagnosis and skeletal survey was performed at 1 year follow-up. Although Patient 2 and Patient 11 presented some elevation in creatinine at diagnosis (1.65 mg/dL and 1.62 mg/dL), the creatinine levels were normalized within 1 month, and creatinine clearance was maintained over 40 mL/min thereafter.

In addition, we cultured BM stromal cells from patients with other plasma cell neoplasms (supplemental Table S2). One patient (Patient 12) had two bone plasmacytomas in the spine and femur without other symptoms and < 10% plasma cells in their BM (plasmacytoma group). Two patients were diagnosed with AL amyloidosis (one with cardiac amyloidosis and the other with renal amyloidosis), and one patient was diagnosed with POEMS syndrome. The percentage of BM plasma cells in patients with AL amyloidosis were 9.2% and 12.5%, and the percentage of plasma cells in patients with POEMS syndrome was 3.6% (supplemental Table S2).

The median age of the patients was 63 years, raging from 40 to 77 years. Nine patients were male, and six were female. The following information was gathered for each patient: date of diagnosis and start of therapy; age; sex; ethnicity; hemoglobin level; level and type of paraprotein; and serum levels of albumin, creatinine, calcium, lactate dehydrogenase (LDH), and β_2_-microglobulin. We also recorded the percentage of BM plasma cell infiltration, conventional cytogenetic results of BM cells by G-banding, and the number of osteolytic lesions. Stage was assessed according to the International Staging System (ISS)^1^ and the Durie-Salmon system.^2^ Most of the MM patients had advanced disease: eight had stage III disease according to the ISS staging system, one had stage II disease, and two had stage I disease.

The control groups were composed of 9 B-cell lymphoma patients with no evidence of BM involvement and 4 patients with mild-to-moderate cytopenia without evidence of hematologic malignancies (nine males and four females). The median age of the control group was 50 years, ranging from 23 to 70 years. The BM samples were collected with informed consent, and the study was reviewed and approved by the Institutional Review Board of Seoul National University College of Medicine.

**Culture and characterization of BM stromal cells**

For all patients, BM aspirates were obtained at the time of initial diagnosis. Buffy coat cells were obtained from BM aspirate samples after centrifugation, and were cultured in RPMI1640 medium (JBI, Seoul, South Korea) supplemented with 10% heat-inactivated fetal bovine serum (FBS; Gibco, Grand Island, NY, USA), 100 units/ml of an antibiotic–antimycotic agent (Gibco), 10 mM HEPES (Gibco), 1 mM sodium pyruvate (Gibco), 4.5 g/L glucose (Gibco) and 0.05 mM 2-mercaptoethanol (Amresco, Solon, OH, USA). After 7 to 10 days in culture, non-adherent cells were removed and the BM stromal cells had visibly adhered to the plasticware (this was considered passage 0; p0).^3^ The culture medium was replaced three times a week until the stromal cell cultures were approximately 70% confluent. When sufficient confluency was reached, the cells were trypsinized (0.05% trypsin-EDTA) and expanded at a 1:2 ratio (p1). Cells were cultured until 5.0 × 10^5^ cells were obtained, up to p8. The cultures were maintained at 37°C with 5% CO_2_.

Selected BM stromal cells from the MM patients (n = 4) and control patients (n = 5) at the final harvest were tested for surface antigen expression using flow cytometry. The following monoclonal antibodies were used to detect hematopoietic cells: anti-CD45-APC (allophycocyanine; Beckman Coulter, Miami, FL, USA), anti-CD34-PerCPCy5.5 (Beckman Coulter) and anti-CD138-PerCPCy5.5 (Beckman Coulter) for the detection of plasma cells and other hematopoietic cells. Antibody combinations including anti-CD44-FITC (fluorescein isothiocyanate), anti-CD29-FITC, anti-CD105-PE (phycoerythrin), anti-CD144-PE, and anti-CD90-PE (all from Becton Dickinson Biosciences Pharmingen, San Jose, CA, USA) were used to characterize BM stromal cells. Antibody-labeled cells were detected on a Navios flow cytometer (Beckman Coulter) and analyzed using Kaluza software (Beckman Coulter).

**Conventional karyotyping by G-banding and fluorescence in situ hybridization (FISH)**

Cytogenetic studies using standard G-banding techniques on heparinized BM aspirate samples were performed as part of the diagnostic work-up. Interleukin-4 (IL-4)-stimulated and unstimulated cultures were generated as described previously.^4^ At least 20 metaphase cells were analyzed whenever possible. Karyotypes were recorded according to the International System for Human Cytogenetic Nomenclature (ISCN) 2013.^5^

Interphase FISH was performed on patient BM aspirate specimens and cultured BM stromal cells to investigate common chromosomal abnormalities known to frequently occur in MM. Commercial FISH probes included an LSI dual-color, break-apart probe for *IGH* translocations; a dual color, dual-fusion translocation probe for t(14;16)(q13;q32)/*IGH-MAF*; a dual color, dual-fusion translocation probe for t(4;14)(p16;q32) /*IGH-FGFR3*; an LSI 13 (RB1) 13q14 probe;an LSI p53 (17p13.1) probe; a Vysis LSI p16 (9p21) SpectrumOrange/CEP 9 SpectrumGreen probe; and an LSI 1p36/1q25 probe (all from Abbott Molecular/Vysis, Des Plaines, IL). Plasma cells in BM aspirates were tested individually using a modification of the simultaneous κ/λ immunoglobulin light chain cytoplasmic staining method (cIg FISH).^6^ Five microliters of a 1:20 dilution of a 1:1 mixture of polyclonal anti-human κ (Clone F0198; DakoCytomation, Glostrup, Denmark) and anti-human λ (Clone F0199; Dako) was added to the samples, which were then incubated for 40 min in the dark in a humidified chamber. Slides were washed twice with phosphate-buffered saline (PBS) and dried. Slides were stained with FISH probes and counter-stained with DAPI, and fluorescence signals were then analyzed on a fluorescence microscope (Zeiss, Göttingen, Germany). Interphase FISH signals were evaluated in 20 plasma cells. The results of the FISH studies were recorded according to the ISCN 2013.

**Gene expression analysis**

Total RNA was extracted using Trizol (Invitrogen Life Technologies, Carlsbad, CA, USA) and purified using RNeasy columns (Qiagen, Valencia, CA, USA) according to the manufacturers’ protocols. After processing with DNase digestion and clean-up procedures, RNA samples were quantified, aliquoted and stored at −80°C until use. For quality control, RNA purity and integrity were evaluated by denaturing gel electrophoresis, OD 260/280 ratio, and analysis on an Agilent 2100 Bioanalyzer (Agilent Technologies, Palo Alto, CA, USA). Total RNA was amplified and purified using the Ambion Illumina RNA amplification kit (Ambion, Austin, TX, USA) to yield biotinylated cRNA according to the manufacturer’s instructions. Briefly, 550 ng of total RNA was reverse-transcribed to cDNA using a T7 oligo(dT) primer. Second-strand cDNA was synthesized, in vitro transcribed, and labeled with biotin-NTP. After purification, the cRNA was quantified using a ND-1000 Spectrophotometer (NanoDrop, Wilmington, DE, USA). Labeled cRNA samples (750 ng) were hybridized to each human HT-12 expression v.4 bead array for 16-18 h at 58°C according to the manufacturer's instructions (Illumina, Inc., San Diego, CA, USA). The array signal was detected using Amersham fluorolink streptavidin-Cy3 (GE Healthcare Bio-Sciences, Little Chalfont, UK) following the bead array manual. Arrays were scanned with an Illumina bead array reader confocal scanner according to the manufacturer's instructions. The microarray data are submitted at Gene Expression Omnibus (GEO).

**Quantitative real-time PCR analysis**

cDNA was produced using the SuperscriptTM RT-PCR System (Invitrogen, Karlsruhe, Germany) according to the manufacturer’s recommendations for oligo(dT)20- primed cDNA synthesis. cDNA synthesis was performed on 500 ng of RNA at 42°C. Finally, cDNA was diluted 1:2 prior to use in the qPCR assay. PCR was performed in an ABI PRISM 7900HT Sequence Detection System (Applied Biosystems, Foster City, CA, USA) in 384-well microtiter plates using a final volume of 10 μL. Optimum reaction conditions were obtained with 5 μL of Universal Master Mix (Applied Biosystems) containing dNUTPs, MgCl_2_, reaction buffer, Ampli Taq Gold, 90 nM of primer(s) and 250 nM of fluorescently labeled TaqMan probe. Finally, 2 μL of template cDNA was added to the reaction mixture. Primer/TaqMan probe combinations were designed for each target sequence. Amplifications were performed starting with a 10-min template denaturation step at 95°C, followed by 40 cycles at 95°C for 15 s and 60°C for 1 min. All samples were amplified in triplicate, and the data were analyzed with Sequence Detector software (Applied Biosystems). We used the comparative cycle threshold (C_T_) method for relative quantification.^7^ The C_T_ value for the gene of interest was measured and normalized to its respective the C_T_ value for GAPDH (the endogenous control).

**Raw data preparation and statistical analyses**

The quality of hybridization and overall chip performance were monitored by visual inspection of the internal quality control checks and the raw scanned data. Raw data were extracted using the software provided by the manufacturer (Illumina GenomeStudio v2009.2 (Gene Expression Module v1.5.4)). Array data were filtered using detection p-values < 0.05 (similar to signal-to-noise) in at least 50% of the samples (we applied a filtering criterion for data analysis, wherein higher signal values were required to obtain a detection p-value < 0.05). The signal of the selected gene was subjected to logarithmic transformation and normalization by the robust spline normalization (RSN) algorithm using a lumi package in Bioconductor software. ^8^ The statistical significance of the expression data was determined using the Significant Analysis of Microarray (SAM) test and fold-change; the null hypothesis was that no difference exists between the two groups.^9^ Hierarchical clustering analysis was performed using complete linkage and Euclidean distance as a measure of similarity. Gene Set Enrichment Analysis (GSEA, Version 2.08) was used to identify a gene set collection of biological processes from an online database available at the GSEA Web site (http://www.broadinstitute.org/gsea) (c2.reactome.v4.0.symbols, and c5.all.v4.0.symbols; gene ontology).^10^ All data analysis and visualization of differentially expressed genes were conducted using R 2.15.2 (www.r-project.org) and SPSS version 17.0 (SPSS Inc., Chicago, IL, USA). *P*-values < 0.05 were considered significant.

**Supplemental Reference**

1. Greipp PR, San Miguel J, Durie BG, Crowley JJ, Barlogie B, Blade J*, et al.* International staging system for multiple myeloma. *Journal of clinical oncology : official journal of the American Society of Clinical Oncology* 2005 May 20; **23**(15)**:** 3412-3420.

2. Durie BG, Salmon SE. A clinical staging system for multiple myeloma. Correlation of measured myeloma cell mass with presenting clinical features, response to treatment, and survival. *Cancer* 1975 Sep; **36**(3)**:** 842-854.

3. Garayoa M, Garcia JL, Santamaria C, Garcia-Gomez A, Blanco JF, Pandiella A*, et al.* Mesenchymal stem cells from multiple myeloma patients display distinct genomic profile as compared with those from normal donors. *Leukemia : official journal of the Leukemia Society of America, Leukemia Research Fund, UK* 2009 Aug; **23**(8)**:** 1515-1527.

4. Hernandez JM, Gutierrez NC, Almeida J, Garcia JL, Sanchez MA, Mateo G*, et al.* IL-4 improves the detection of cytogenetic abnormalities in multiple myeloma and increases the proportion of clonally abnormal metaphases. *British journal of haematology* 1998 Oct; **103**(1)**:** 163-167.

5. International Standing Committee on Human Cytogenetic Nomenclature, Shaffer LG, McGowan-Jordan J, Schmid M. *ISCN 2013: An International System for Human Cytogenetic Nomenclature (2013)*. Karger: Basel ; Unionville, CT, 2013.

6. Ahmann GJ, Jalal SM, Juneau AL, Christensen ER, Hanson CA, Dewald GW*, et al.* A novel three-color, clone-specific fluorescence in situ hybridization procedure for monoclonal gammopathies. *Cancer genetics and cytogenetics* 1998 Feb; **101**(1)**:** 7-11.

7. Pfaffl MW. A new mathematical model for relative quantification in real-time RT-PCR. *Nucleic acids research* 2001 May 1; **29**(9)**:** e45.

8. Du P, Kibbe WA, Lin SM. lumi: a pipeline for processing Illumina microarray. *Bioinformatics (Oxford, England)* 2008 Jul 1; **24**(13)**:** 1547-1548.

9. Tusher VG, Tibshirani R, Chu G. Significance analysis of microarrays applied to the ionizing radiation response. *Proceedings of the National Academy of Sciences of the United States of America* 2001 Apr 24; **98**(9)**:** 5116-5121.

10. Subramanian A, Tamayo P, Mootha VK, Mukherjee S, Ebert BL, Gillette MA*, et al.* Gene set enrichment analysis: a knowledge-based approach for interpreting genome-wide expression profiles. *Proceedings of the National Academy of Sciences of the United States of America* 2005 Oct 25; **102**(43)**:** 15545-15550.

**Supplemental Figure Legend**

**Supplemental Figure S1.** Gene expression levels of *NOTCH2NL* (A and B), *IGLL1* (C and D), and *STNM2* (E and F) among patients with multiple myeloma (MM) and other plasma cell neoplasms, which were estimated by (A, C, and E) microarray and (B, D, and F) qPCR assay.
