# Supplementary material for: Bone marrow stromal cells show distinct gene expression patterns depending on symptomatically involved organs in multiple myeloma
Source: Blood Cancer J. 2016 Sep 23;6(9):e476–. doi: 10.1038/bcj.2016.86 (PMC5056976; doi:10.1038/bcj.2016.86)
Supplement: Supplementary Tables [file bcj201686x2.doc]

**Supplemental Tables**

**Supplemental Table S1.** Characteristics of 11 patients with plasma cell myeloma

| **Patient** | **Age** | **Sex** | **Clinical presentation** | **ISS** | **DSS** | **Bone lesion** | **BM PC (%)** | **Ig** | **M-protein (g/L)** | **Cr (mg/dL)** | **Ca (mg/dL)** | **Hgb (g/dL)** | **Alb (g/dL)** | **β2m (mg/L)** | **LDH (mg/L)** |
| --- | --- | --- | --- | --- | --- | --- | --- | --- | --- | --- | --- | --- | --- | --- | --- |
| 1 | 55 | M | Asymptomatic | I | IA | − | 10.0 | Gλ | 12.5 | 0.93 | 9.7 | 14.3 | 4.5 | 2.05 | 242 |
| 2 | 73 | M | Bone lesions | III | IIIA | ++ | 33.3 | Gκ | 87.5 | 1.65 | 10.0 | 7.1 | 2.6 | 9.12 | 107 |
| 3 | 68 | F | Bone lesions, Renal impairment | III | IIIB | ++ | 59.1 | κ | 2.7 | 2.55 | 10.6 | 7.3 | 3.2 | 11.89 | 237 |
| 4 | 62 | F | Bone lesions | III | IIIA | ++ | 58.0 | Aκ | 68.9 | 1.04 | 10.0 | 7.4 | 2.9 | 6.58 | 1335 |
| 5 | 54 | F | Bone lesions | III | IIIA | ++ | 64.1 | Gλ | 65.6 | 0.80 | 9.0 | 7.4 | 10.7 | 6.08 | 250 |
| 6 | 59 | M | Bone lesions | III | IIIA | ++ | 83.4 | λ | 32.7 | 1.80 | 10.6 | 9.0 | 4.4 | 5.95 | 199 |
| 7 | 73 | M | Bone lesions | I | IIA | ++ | 25.7 | Gκ | 0.3 | 0.78 | 10.7 | 14.4 | 4.1 | 2.52 | 247 |
| 8 | 71 | M | Renal impairment | III | IIB | − | 66.1 | λ | 5.3 | 6.91 | 9.9 | 8.1 | 4.1 | 18.36 | 217 |
| 9 | 40 | M | Renal impairment | III | IIB | − | 26.6 | λ | 9.2 | 11.10 | 9.0 | 9.4 | 3.7 | 23.35 | 296 |
| 10 | 71 | M | Renal impairment | II | IIB | − | 11.0 | Aλ | 10.1 | 2.80 | 8.2 | 8.0 | 2.5 | 3.18 | NA |
| 11 | 77 | F | Anemia | III | IIIA | +/− | 43.5 | Gκ | 51.4 | 1.62 | 8.9 | 8.2 | 3.3 | 9.87 | 228 |

Abbreviations: Alb, albumin; BM, bone marrow; β2m, β2 microglobulin; Cr, creatinine; Ca, calcium; DSS, Durie and Salmon stage; F, female, Hgb, hemoglobin; Ig, immunoglobulin; ISS, international staging system for multiple myeloma; LDH, lactate dehydrogenase; M, male; NA, not assessed; PC, plasma cell; BM PC, percentages of plasma cells counted in bone marrow aspiration slides.

**Supplemental Table S2.** Characteristics of 4 patients with other plasma cell neoplasms

| **Patient** | **Age** | **Sex** | **Clinical presentation** | **ISS** | **DSS** | **Bone lesion** | **BM PC (%)** | **Ig** | **M-protein (g/L)** | **Cr (mg/dL)** | **Ca (mg/dL)** | **Hgb (g/dL)** | **Alb (g/dL)** | **β2m (mg/L)** | **LDH (mg/L)** |
| --- | --- | --- | --- | --- | --- | --- | --- | --- | --- | --- | --- | --- | --- | --- | --- |
| 12 | 61 | M | Plasmacytoma | I | IIA | + | 8.6 | Aκ | 2.7 | 0.84 | 9.1 | 10.8 | 4.1 | 1.59 | 151 |
| 13 | 63 | M | AL amyloidosis | II | IIA | − | 12.5 | Gλ | 16.8 | 0.98 | 9.7 | 11.8 | 3.5 | 3.68 | 486 |
| 14 | 64 | F | AL amyloidosis | NA | IIB | − | 9.2 | κ | 0.2 | 2.19 | 8.5 | 9.0 | 2.6 | NA | 289 |
| 15 | 48 | F | POEMS syndrome | NA | IIA | − | 3.6 | Aλ | 7.4 | 1.07 | 8.6 | 15.0 | 4.1 | NA | 187 |

Abbreviations: Alb, albumin; BM, bone marrow; β2m, β2 microglobulin; Cr, creatinine; Ca, calcium; DSS, Durie and Salmon stage; F, female, Hgb, hemoglobin; Ig, immunoglobulin; ISS, international staging system for multiple myeloma; LDH, lactate dehydrogenase; M, male; NA, not assessed; PC, plasma cell; BM PC, percentages of plasma cells counted in bone marrow aspiration slides.

**Supplemental Table S3.** Growth culture characteristics of bone marrow stromal cells.

|  | **Control**  **(n = 13)** | **Plasma cell neoplasm**  **(n = 15)** | **Bone lesion group**  **(n = 6)** | **Renal failure group**  **(n = 3)** | **Other groups**  **(n = 6)** |
| --- | --- | --- | --- | --- | --- |
| Days in culture | 50 (24-64) | 43 (22-110) | 62 (42-110) | 41 (22-53) | 40 (24-79) |
| Passage | 6 (4-8) | 6 (5-8) | 6 (5-8) | 6 (6-7) | 6 (5-7) |
| Final cell counts (× 10^5^ cells) | 10.7 (3.0-1002.6) | 5.7 (1.3-91.8) | 14.4 (1.3-91.8) | 6.9 (4.8-16.9) | 10.0 (4.1-14.5) |

The data are presented as the median (range).

**Supplemental Table S4.** Cytogenetic characteristics of bone marrow plasma cells and stromal cells

| **Patient** | **Group** | **BM plasma cells** | |  | **BM stromal cells** |
| --- | --- | --- | --- | --- | --- |
|  |  | **G-banding** | **FISH** |  | **FISH** |
| 1 | asymptomatic | 46,XY[20] | Trisomy 9: 90% |  | Normal |
| 2 | bone | 46,XY[20] | Normal |  | Normal |
| 3 | bone (mixed) | 46,XX[7] | del *RB1*: 100% |  | Normal |
| 4 | bone | 46,XX,+1,dic(1;22)(p11;p11.1),der(2)t(2;8)(p12;q24),der(8)del(8)(p21)t(2;8),+9,-11,del(11)(q23),-12,-13,+15,der(17)t(1;17)(q12;p13),der(20)t(19;20)(p13.1;p13),der(?)t(?;11)(?;q13)[7]/46,XX[2] | t(4;14): 95%; del *RB1*: 100%; Trisomy 9: 100%; dup(1q): 100% |  | Normal |
| 5 | bone | 46,XX[8] | t(4;14): 88%; del *RB1*: 100%; dup(1q): 100% |  | Normal |
| 6 | bone | 46,X,der(?Y;6)(q10;p10),t(2;17)(p10;p10),t(3;10)(p13;p13),der(6)del(6)(q15q21)?inv(6)(q21q25),t(11;14)(q13;q32)[8]/47,sl,+der(3)t(3;10)[2]/47,sl,+18[3]/46,XY[4] | *IGH* rearrangement: 100%; trisomy 17: 100% |  | Normal |
| 7 | bone | 46,XY[20] | Normal |  | Normal |
| 8 | renal | 46,XY[8] | *IGH* rearrangement: 100%; del *RB1*: 98%; dup(1q): 100% |  | Normal^*^ |
| 9 | renal | 46,XY,+1,der(1;16)(q10;p10),t(11;14)(q13;q32),add(19)(q13.3)[8]/46,XY[12] | *IGH* rearrangement: 100%; dup(1q): 100% |  | Normal |
| 10 | renal | 46,XY[20] | Normal |  | NA |
| 11 | anemia | 46,XX,1cenh+,der(3)t(3;19)(q21;p13.1),-14,?del(16)(q24),-19,+22,der(?)t(?;3)(?;q21)[6]/46,XX[16] | Normal |  | NA |
| 12 | Plasmacytoma | 47,XY,+der(1;19)(q10;p10)X2,add(6)(q13),add(11)(p15),-13,add(19)(q13.3)[4]/46,XY[16] | *IGH* rearrangement: 50%; del *RB1*: 60%; dup(1q): 50% |  | Normal |
| 13 | AL amyloidosis | 46,XY[20] | Normal |  | NA |
| 14 | AL amyloidosis | 46,XX[20] | Normal |  | NA |
| 15 | POEMS | 46,XX[21] | Normal |  | NA |

Abbreviations: BM, bone marrow; FISH, fluorescence in situ hybridization; NA, not assessed.

^*^For this sample, a minor population of tetraploidy (5%) was identified in chromosome 1; however, this observation was considered non-specific.
