# Supplementary figures and images for: Bone marrow stromal cells show distinct gene expression patterns depending on symptomatically involved organs in multiple myeloma
Source: Blood Cancer J. 2016 Sep 23;6(9):e476–. doi: 10.1038/bcj.2016.86 (PMC5056976; doi:10.1038/bcj.2016.86)

**A**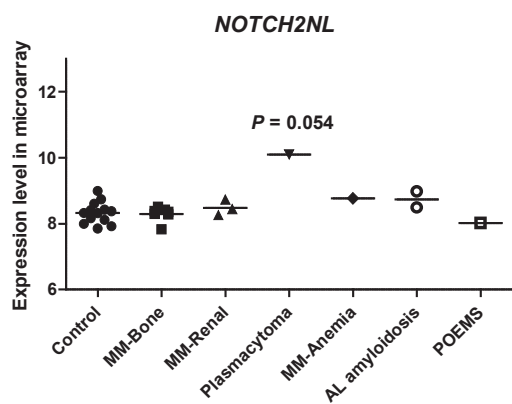**B**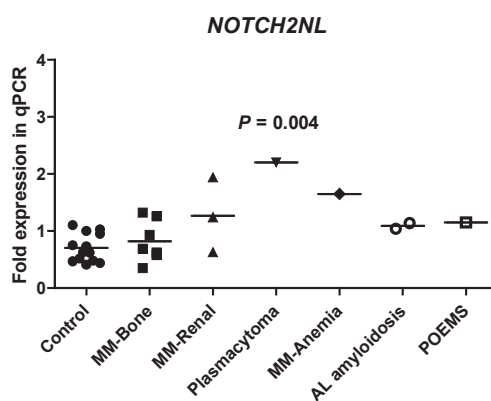**C**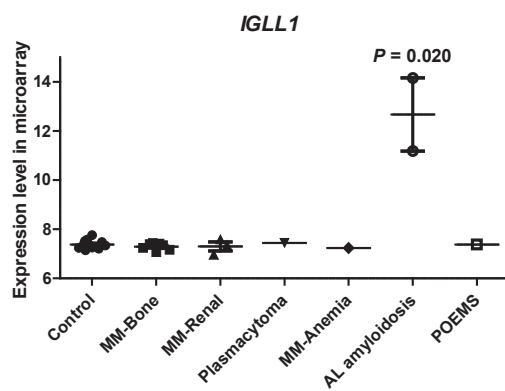**D**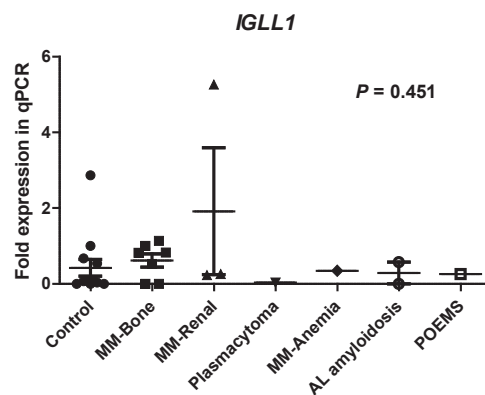**E**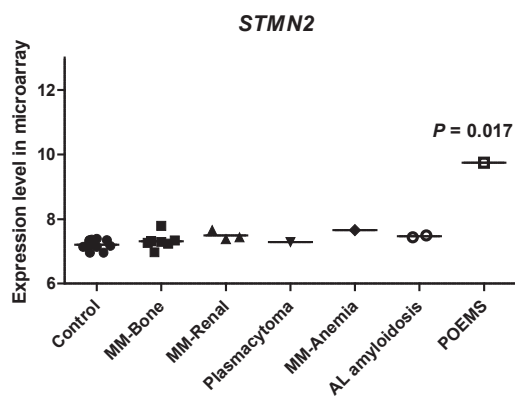**F**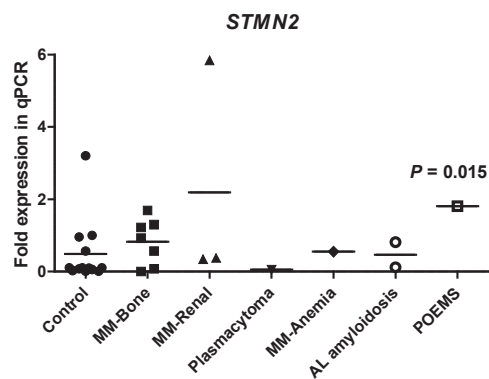

**Supplemental Figure S1**

Supplement: Supplementary Figure S1 [file bcj201686x3.pdf]
